# Integrating Electronic Health Records and Polygenic Risk to Identify Genetically Unrelated Comorbidities of Schizophrenia That May Be Modifiable

**DOI:** 10.1016/j.bpsgos.2024.100297

**Published:** 2024-02-28

**Authors:** Tess Vessels, Nicholas Strayer, Hyunjoon Lee, Karmel W. Choi, Siwei Zhang, Lide Han, Theodore J. Morley, Jordan W. Smoller, Yaomin Xu, Douglas M. Ruderfer

**Affiliations:** a Division of Genetic Medicine, Department of Medicine, Vanderbilt University Medical Center, Nashville, Tennessee; b Center for Digital Genomic Medicine, Department of Medicine, Vanderbilt University Medical Center, Nashville, Tennessee; c Vanderbilt Genetics Institute, Vanderbilt University Medical Center, Nashville, Tennessee; d Department of Biostatistics, Vanderbilt University Medical Center, Nashville, Tennessee; e Psychiatric & Neurodevelopmental Genetics Unit, Center for Genomic Medicine, Massachusetts General Hospital, Boston, Massachusetts; f Center for Precision Psychiatry, Department of Psychiatry, Massachusetts General Hospital, Boston, Massachusetts; g Department of Biomedical Informatics, Vanderbilt University Medical Center, Nashville, Tennessee; h Department of Psychiatry and Behavioral Sciences, Vanderbilt University Medical Center, Nashville, Tennessee

**Keywords:** Comorbidities, Electronic health records, Genetics, Genomics, Schizophrenia, Statistics

## Abstract

**Background:**

Patients with schizophrenia have substantial comorbidity that contributes to reduced life expectancy of 10 to 20 years. Identifying modifiable comorbidities could improve rates of premature mortality. Conditions that frequently co-occur but lack shared genetic risk with schizophrenia are more likely to be products of treatment, behavior, or environmental factors and therefore are enriched for potentially modifiable associations.

**Methods:**

Phenome-wide comorbidity was calculated from electronic health records of 250,000 patients across 2 independent health care institutions (Vanderbilt University Medical Center and Mass General Brigham); associations with schizophrenia polygenic risk scores were calculated across the same phenotypes in linked biobanks.

**Results:**

Schizophrenia comorbidity was significantly correlated across institutions (*r* = 0.85), and the 77 identified comorbidities were consistent with prior literature. Overall, comorbidity and polygenic risk score associations were significantly correlated (*r* = 0.55, *p* = 1.29 × 10^−118^). However, directly testing for the absence of genetic effects identified 36 comorbidities that had significantly equivalent schizophrenia polygenic risk score distributions between cases and controls. This set included phenotypes known to be consequences of antipsychotic medications (e.g., movement disorders) or of the disease such as reduced hygiene (e.g., diseases of the nail), thereby validating the approach. It also highlighted phenotypes with less clear causal relationships and minimal genetic effects such as tobacco use disorder and diabetes.

**Conclusions:**

This work demonstrates the consistency and robustness of electronic health record–based schizophrenia comorbidities across independent institutions and with the existing literature. It identifies known and novel comorbidities with an absence of shared genetic risk, indicating other causes that may be modifiable and where further study of causal pathways could improve outcomes for patients.

## Plain Language Summary

Patients with schizophrenia have many co-occurring diseases that contribute substantially to premature mortality of 10 to 20 years. Conditions that are comorbid but lack shared genetic risk with schizophrenia are likely to have causes that are more modifiable. Here, we calculated comorbidity from electronic health records from 2 independent health care institutions and associations with schizophrenia polygenic risk scores across the same phenotypes in linked biobanks. We identified known and novel diseases comorbid with schizophrenia, thereby validating our approach.

Patients with schizophrenia have a reduced life expectancy of 10 to 20 years (1, 2, 3, 4). Contributing factors include smoking, substance abuse, medication side effects, poor health maintenance, accidents, and suicide (2,3,5). However, medical comorbidities account for 70% of the premature mortality experienced by patients with schizophrenia (6,7). The mortality ratio for schizophrenia increases almost 4-fold when comorbid with other conditions (4,8) and continues to increase with additional comorbidities (4). Multimorbidity (2 or more comorbidities) is common, occurring in 64% of patients with schizophrenia (6). Underdiagnosis of comorbid conditions and delays in seeking medical care are important factors contributing to early mortality (7). Schizophrenia has been associated with a wide variety of comorbidities including cancer and diseases affecting most major organ systems such as endocrine, metabolic, respiratory, cardiovascular, digestive, neurological, musculoskeletal, genitourinary, and immunological (2,6,9, 10, 11, 12, 13, 14, 15, 16, 17, 18, 19, 20, 21, 22, 23, 24, 25, 26, 27, 28, 29, 30, 31, 32).

Schizophrenia is a highly heritable disorder that shares significant genetic risk with multiple other phenotypes (33,34). Multiple recent studies have leveraged biobanks paired with electronic health record (EHR) data to evaluate comorbidities associated with schizophrenia polygenic risk scores (PRSs) (35, 36, 37). The first study combined data from 4 independent health care systems to assess the relationship between genetic risk of schizophrenia and other phenotypes (35). Using a schizophrenia PRS, they performed a phenome-wide association study (PheWAS) at each site and combined the results in a cross-site meta-analysis. The study identified associations of the schizophrenia PRS with psychiatric conditions, neurological disorders, obesity, urinary syndromes, viral hepatitis, synovitis and tenosynovitis, and malaise and fatigue (35). A second study explored the significance and consistency of schizophrenia PRS with other phenotypes across the network of hospitals in the U.S. Veterans Affairs Hospital System (37). Genetic risk of schizophrenia has also recently been linked with poor cardiac phenotypes (36). These studies demonstrate the power of biobanks linked to EHRs for identifying phenotypes that have a shared genetic risk with schizophrenia.

Comorbidity or phenotypic correlation is often a byproduct of shared underlying genetic risk or genetic correlation (38). Therefore, it is expected that most schizophrenia comorbidities will share genetic risk with schizophrenia as seen in the studies described above. However, when there is a lack of shared genetic etiology for a comorbidity, another cause is likely. These comorbidities are more likely to be driven by adverse events from treatment, unhealthy behavior, or environmental factors as opposed to innate biological risk and therefore should be enriched for modifiable associations (39). Knowing this provides opportunities to intervene regardless of patients’ genetic risk for schizophrenia and potentially reduces the burden of comorbidities on their life expectancy. Novel approaches to identifying this set of comorbidities could lead to improved causal understanding and preventive efforts to reduce these risks. Leveraging EHR data linked to biobanks can provide a direct path to identifying and comparing comorbidities with and without genetic association to implicate potentially modifiable comorbidities.

Here, we assessed comorbidity using EHR billing codes, adjusting for demographic information and total illness in 2 health care systems, and validated our findings with prior literature. Associations of the schizophrenia PRS with the same set of codes across 2 linked biobanks were used to identify phenotypes with shared schizophrenia genetic risk. Then, we applied equivalence testing to identify comorbidities that had a significant absence of association with the schizophrenia PRS (i.e., statistical evidence of absence). These phenotypes represent opportunities to identify other, potentially modifiable causes of these comorbidities.

## Methods and Materials

### Quantifying the Degree of Comorbidity With Schizophrenia Using Phecodes From 2 Health Care Systems

Demographic information including age, coded sex, coded race, and ICD billing codes were obtained for 250,000 randomly chosen people from the EHRs of 2 major health care systems, Vanderbilt University Medical Center (VUMC) and Mass General Brigham (MGB). A total of 250,000 patients were used to balance a representative sample with computational burden and cost. Individuals were selected when all data were available, so there were no missing covariates. Coded sex and coded race were treated as categorical variables, including unknown or missing status in the regression model. MGB included both ICD-9 and -10 codes while VUMC used ICD-9 codes only. There was no cross-mapping between ICD-9 and -10. Instead, the PheWAS package in R was used to consolidate the ICD codes and their counts into phecodes (definition 1.2) (40, 41, 42). Phecodes listed as exclusions (conditions that are similar to the phecode of interest) were removed from the controls (https://phewascatalog.org/phecodes) (43). Codes were consolidated across all encounter types for the entire length of the individual’s record. Genetic associations using phecodes have been found to be highly reproducible across multiple biobanks (44).

Pairwise logistic regressions were performed on the set of patients for each health care system separately across all phecodes, of which 1701 phecodes occurred in at least one patient with schizophrenia. More specifically, the occurrence of a phecode (phecode_b) depending on the presence of another phecode (phecode_a) was estimated as well as the reverse relationship (i.e., phecode_a depending on the presence of phecode_b), which resulted in 1701 × 1701 or 2,893,401 regressions. For our purposes, phecode_b would be the 295.1 phecode for schizophrenia, so that it was both the independent and dependent variable in the regressions at each site. A person was considered a case for a phenotype if 2 or more phecodes were present and a control if no phecodes were present. Patients with a single instance of a phecode were treated as missing. Schizophrenia cases were defined by the presence of at least 2 independent instances of the 295.1 phecode. Covariates included current age, age at last visit (EHR age), coded sex (male, female, or unknown), inferred or self-reported ethnicity (Asian, Black, Native American/Alaskan, Other, Pacific Islander, White, or unknown), and the log of the number of unique phecodes per patient to account for the likelihood of seeing comorbidity as a product of an individual’s health record. The results from the regressions at each health care system were then combined into 1 comorbidity measure by taking the weighted average of the *Z* scores. This was done according to the following equation: ${\text{Comorbidity}}_{A,B}^{V}$ is the combined *Z* score from the pairwise logistic regressions at VUMC; ${\text{comorbidity}}_{A,B}^{M}$ is the combined *Z* score from the pairwise logistic regressions at MGB; and $N_{A,B}^{V}$ and $N_{A,B}^{M}$ are the number of shared patients in the VUMC and MGB cohorts, respectively.

(1) $${\text{Comorbidity}}_{A,B}^{C}\;=\;\frac{\left({\text{Comorbidity}}_{A,B}^{V}\;\cdot \;N_{A,B}^{V}\right)\;+\;\left({\text{Comorbidity}}_{A,B}^{M}\;\cdot \;N_{A,B}^{M}\right)}{N_{A,B}^{V}\;+\;N_{A,B}^{M}}$$

The *p* values for these clinical associations were estimated using these weighted *Z* scores and the following formula: *p* = 2 × pnorm(−abs(*Z* score)) and then Bonferroni corrected for multiple testing.

### Schizophrenia Comorbidity Literature Review

A literature review was conducted to evaluate agreement between the EHR-identified comorbidities and what is known from the previous literature. We searched the published literature as of December 11, 2023 for described comorbidities of schizophrenia. We used medical subject headings or MeSH terms “schizophrenia” and “comorbidity” or “multimorbidity” to search PubMed for relevant papers. The search was restricted to papers from 2011 to December 11, 2023 where the full text was available in English, which resulted in 915 papers. We retained only case/control studies, retrospective cohort studies, review articles, and meta-analyses and removed 108 papers that were clinical trials, case reports, or letters to the editor. Requiring that the papers detailed comorbid relationships with individual disorders excluded an additional 763 papers. These papers often addressed comorbidities as a group among specific circumstances (e.g., COVID-19 pandemic, imaging data for volumetric differences in gray matter, postsurgery period, or health care utilization). In total, 44 papers remained, and the results section of each was reviewed for any mention of a comorbid relationship with schizophrenia. Among this set, the phenotypes mentioned in at least 2 papers were included. There was a total of 72 comorbidities for schizophrenia from these papers, 59 of which mapped to a unique phecode. The other 13 comorbidities did not map for lack of or too much specificity (e.g., cancer vs. malignant neoplasm of the thoracic esophagus). A hypergeometric test was performed to determine whether the overlap of literature-based comorbidities and EHR-based comorbidities was greater than that expected by chance.

### Genotype Sample Description, Ancestry Estimation, and Quality Control

Genotyping quality control and imputation were performed individually at both institutes. The 250,000 patients used for estimating comorbidity were selected randomly. Therefore, those who also had genotyped information were a small, random subset of the entire genotyped cohort used for the PRS analyses. Similar filtering steps were applied to both genotype sets such as excluding single nucleotide polymorphisms (SNPs) based on missingness rate, minor allele frequency, Hardy-Weinberg equilibrium, and strand ambiguity. Individuals were removed based on sex discordance, excess heterozygosity, or relatedness. Principal components of ancestry were estimated and compared with the 1000 Genomes phase 3 dataset as the reference for inferring ancestry (45). Ancestry was assigned at VUMC using K nearest neighbors and at MGB using a random forest classifier. Detailed sample descriptions, quality control, and ancestry estimation are provided for each site separately in Supplemental Methods in Supplement 1.

### PRS Calculation and PheWAS

The PRS was calculated to find genetic associations with other clinical phenotypes using the Python-based program PRS-CS (46). PRS-CS is a Bayesian regression approach that uses continuous shrinkage priors for SNP effect sizes and the 1000 Genomes Project European cohort to represent the linkage disequilibrium structure between SNPs (46). The SNP effect sizes from the PGC’s (Psychiatric Genomic Consortium) latest available genome-wide association study (GWAS) for schizophrenia were used to calculate the PRS (47). These results came from a meta-analysis of 90 cohorts that included 67,390 cases and 94,015 controls of European and East Asian ancestries as well as 7386 cases and 7008 controls of African American and Latino ancestries, resulting in 263 genome-wide significant loci from the initial 7,585,078 SNPs analyzed (47). For European ancestries specifically, there were 53,386 cases and 77,258 controls (47). The scaling parameter, phi, was set to 1 × 10^−2^ to represent the highly polygenic architecture of schizophrenia (48). The PRS-CS–generated posterior effects for SNPs were then used to calculate a PRS for each cohort using the PLINK1.9–score flag, with sum modification to output SCORESUM instead of SCORE (49). PheWAS analysis was conducted in 2 ways: with patients with schizophrenia included and excluded from the PRS estimation (Figures S3 and S7 in Supplement 1, respectively). There were no major differences in significant associations whether schizophrenia cases were or were not included.

For each of the phecodes with at least one person as a case (*n* = 1768 VUMC; *n* = 1639 MGB; *n* = 1586 combined), we performed a logistic regression to test its association with the schizophrenia PRS including covariates of sex, current age, and the first 10 principal components of ancestry within the European population where sample size was sufficient. We also included record length as a covariate, which was calculated as the difference in days between the first and last diagnostic code.

As above, at least 2 instances of the phecode had to be present to be considered a case, and phecodes listed as exclusions according to the phewas catalog were removed from the controls (43). The cross-site information was combined in a fixed-effect inverse variance-weighted meta-analysis using the phewasMeta() function included in the PheWAS package (41). To maximize the utility of the PRS in the African ancestries sample (*n* = 12,024), PRS-CSx was used (see Supplemental Methods in Supplement 1).

### Statistical Testing for the Absence of Difference in Distribution of PRS Between Comorbid Cases and Controls

The two one-sided tests (TOST) procedure was used to statistically identify which comorbidities showed a significant absence of PRS association. Specifically, the equivalence test was used to assign statistical significance to how similar the distributions of the schizophrenia PRS were between cases and controls for each of the 77 significant EHR comorbidities. The tost() function from the TOSTER package version 0.4.1 in R was used to perform TOST of the PRS distributions between cases and controls (50). A Cohen’s *d* of 0.2 and −0.2 were used as the equivalence bounds. The TOST procedure was performed at each site independently. The *p* values from the TOST procedure were Bonferroni corrected for multiple comparisons based on the number of phecodes tested at both sites and with meta-analysis PRS results (*n* = 65). These phenotypes were meta-analyzed according to Fisher’s method for combining *p* values to estimate χ^2^ values and then converted to *p* values using the pchisq() function in R; then the Bonferroni correction was applied for the shared 65 phenotypes. To isolate phecodes that did not share genetic risk with schizophrenia, we reduced this set of phenotypes to include only those that were nonsignificant for the meta-analysis of the schizophrenia PRS.

## Results

### Defining EHR-Based Comorbidities of Schizophrenia

Pairwise logistic regressions of billing codes grouped into 1701 phecodes were performed, accounting for demographic information and total illness, on clinical data from 2 health care systems (VUMC and MGB) to quantify comorbidity (see Methods and Materials; Table S1 in Supplement 2). For all pairs of phecodes, we observed significant correlation (*r* = 0.79, Pearson) of comorbidity (*Z* scores) across sites. For comorbidity with schizophrenia, the correlation across sites was stronger (*r* = 0.85, Pearson). Given the consistency across institutions, we calculated a combined comorbidity measure for each phecode pair using the weighted average of the *Z* scores across VUMC and MGB. This metric was used as the primary comorbidity measure for clinical outcomes in all subsequent analyses.

**Figure 1:**
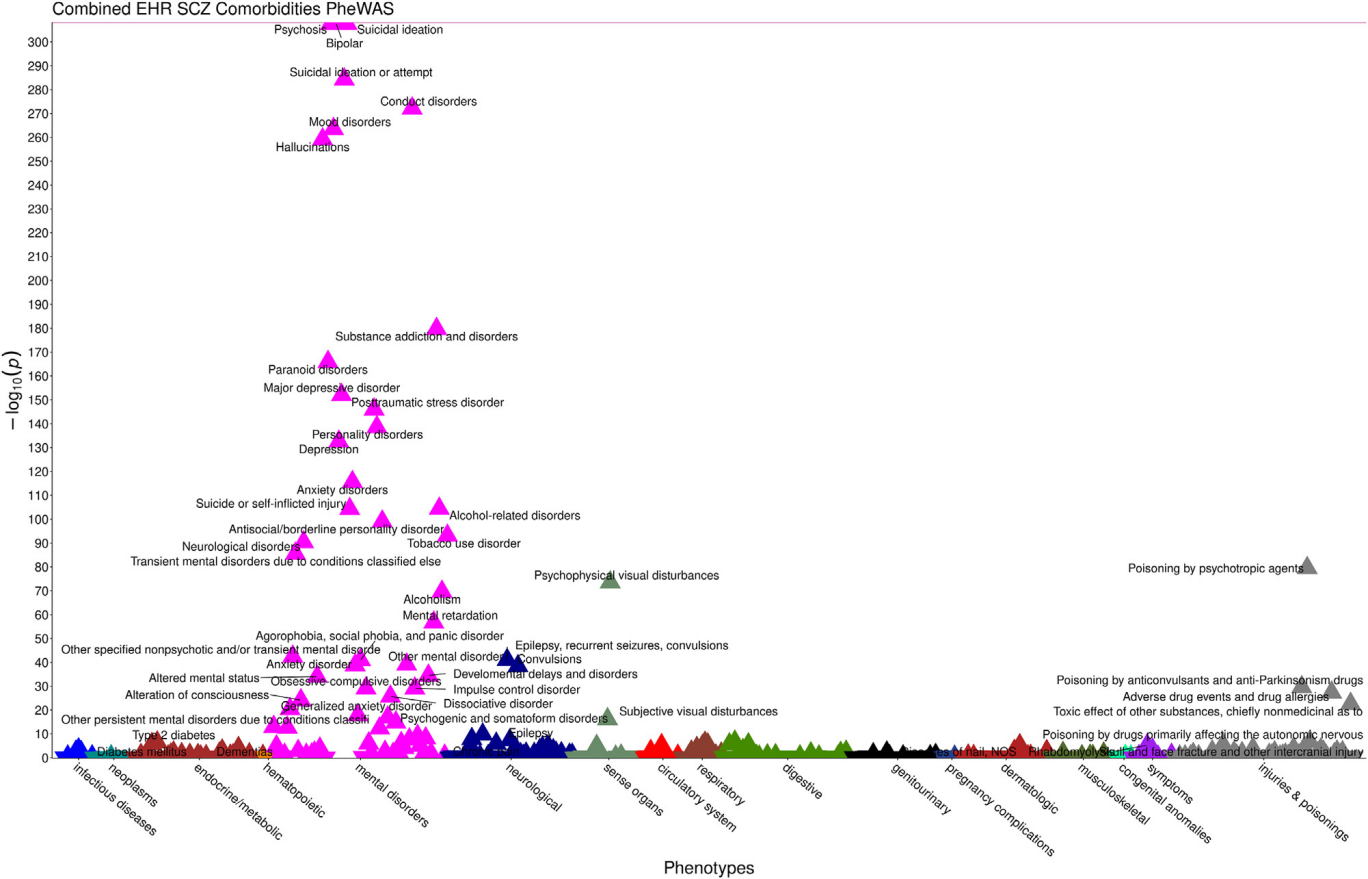
Bonferroni-significant electronic health record (EHR)–based schizophrenia (SCZ) comorbidities across both health care systems. The x-axis is phecodes grouped by category. The y-axis is the negative log_10_ of the *p* values for the phenotypes associated with schizophrenia from the regressions. Labeled triangles represent phenotypes that showed significant association after Bonferroni correction, with either positive (upright triangle) or negative (inverted triangle) direction of effect. NOS, not otherwise specified; PheWAS, phenome-wide association study.

After Bonferroni correction for 1701 tests (*p* < 2.94 × 10^−5^), there were 77 significant phenotypes that were comorbid with schizophrenia (Figure 1). Forty-six of these were psychiatric phenotypes including psychosis, suicidal ideation, bipolar disorder, suicidal ideation or attempt, conduct disorders, mood disorders, and hallucinations. Thirty-one were other medical codes including codes for 7 neurological diseases: epilepsy, recurrent seizures, convulsions; torsion dystonia; convulsions; extrapyramidal disease and abnormal movement disorders; chronic pain; epilepsy; and other and unspecified disorders of the nervous system. The second-largest group of nonpsychiatric comorbidities was injuries and poisonings: poisoning by psychotropic agents; poisoning by anticonvulsants and anti-Parkinsonism drugs; adverse drug events and drug allergies; toxic effect of other substances, chiefly nonmedicinal as to source; poisoning by drugs primarily affecting the autonomic nervous system; and skull and face fracture and other intercranial injury. A complete list of the significant phenotypes for the combined, VUMC only, and MGB only EHR comorbidities are reported in Table S2 in Supplement 2 and Figures S1 and S2 in Supplement 1 (51).

### EHR-Based Comorbidities Show Consistency With Comorbidities Described in the Literature

To assess the robustness of the EHR-based schizophrenia comorbidities, we compared our results to those described in the literature. A comprehensive literature review identified 44 papers that implicated specific phenotypes that are significantly comorbid with schizophrenia. Among these papers, there were 40 medical and 19 psychiatric comorbidities that were identified in 2 or more of the papers and matched to the closest specific phecodes in our data (Table 1). Among the 40 medical phenotypes, 7 were significant and had a positive effect size in our EHR data. Of the 19 psychiatric phenotypes, 18 were significant with a positive effect size. When combined, 25 of 59 comorbidities replicated based on EHR comorbidities, which was significantly (*p* = 2.35 × 10^−12^) more than that expected due to chance based on a hypergeometric test. Overlap among the medical phenotypes (*p* = 5.54 × 10^−3^) and the psychiatric phenotypes (*p* = 4.12 × 10^−27^) were also independently significant. There were 45 phenotypes significantly associated with schizophrenia in the EHR but without multiple mentions across the papers; most were among the psychiatric, neurological, and injuries and poisoning categories (Table S3 in Supplement 2).

**Table 1:** Comparison of Schizophrenia Comorbidities in the Literature to Our EHR-Based Comorbidities

Phenotype	Phecode	Phecode Description	Category	Combined EHR *p* Value	Combined *Z* Score	Combined Overlap *N*	References
Bipolar Disorder	296.1	Bipolar	Mental disorders	<2.3 × 10^−308^	47.9	877	(9,27,52)
Suicide (Attempts)	297	Suicidal ideation or attempt	Mental disorders	5.10 × 10^−285^	36.1	568	(2,12,31,52, 53, 54)
Mood/Affective Disorders	296	Mood disorders	Mental disorders	2.91 × 10^−264^	34.7	1463	(52,55,56)
Substance Use Disorder	316	Substance addiction and disorders	Mental disorders	1.76 × 10^−180^	28.6	625	(15,27,28,32,52,53,57, 58, 59)
Cannabis Use Disorder	316	Substance addiction and disorders	Mental disorders	1.76 × 10^−180^	28.6	625	(12,57, 58, 59)
Opioid Abuse	316	Substance addiction and disorders	Mental disorders	1.76 × 10^−180^	28.6	625	(12,57,58)
Delusional Disorders or Other Nonorganic Psychoses	295.2	Paranoid disorders	Mental disorders	1.27 × 10^−166^	27.5	196	(27,55,59)
Posttraumatic Stress Disorder	300.9	Posttraumatic stress disorder	Mental disorders	1.1 × 10^−146^	25.8	324	(52,57)
Personality Disorder	301	Personality disorders	Mental disorders	2.67 × 10^−139^	25.1	207	(52,55,59)
Depression	296.2	Depression	Mental disorders	2.93 × 10^−133^	24.6	1033	(9,12,27,28,32,52,53)
Anxiety Disorders	300	Anxiety disorders	Mental disorders	2.52 × 10^−116^	22.9	990	(9,12,27,28,52,59)
Alcohol Use Disorders	317	Alcohol-related disorders	Mental disorders	3.87 × 10^−105^	21.8	442	(11,12,15,27,28,32,52,57,58,60)
Antisocial Personality Disorder	301.2	Antisocial/borderline personality disorder	Mental disorders	8.8 × 10^−100^	21.2	121	(52,59)
Tobacco Substance Abuse/Smoking	318	Tobacco use disorder	Mental disorders	7.68 × 10^−94^	20.6	671	(11,24)
Neurological Disorders	292	Neurological disorders	Mental disorders	4.06 × 10^−91^	20.2	675	(2,4,6,13,19,29,31,61)
Organic Mental Disorder	291.1	Transient mental disorders due to conditions classified elsewhere	Mental disorders	2.66 × 10^−86^	19.7	68	(28,55)
Intellectual Disabilities	315.3	Mental retardation	Mental disorders	1.66 × 10^−57^	16	130	(28,55,56)
Viral Hepatitis	70	Viral hepatitis	Infectious diseases	2.53 × 10^−14^	7.6	160	(2,20)
Epilepsy	345.1	Epilepsy	Neurological	4.09 × 10^−8^	5.5	123	(2,11,12,20,62)
HIV Infection	71	HIV disease	Infectious diseases	4.60 × 10^−8^	5.5	55	(2,20,28)
Chronic Pain	338.2	Chronic pain	Neurological	1.12 × 10^−6^	4.9	168	(14,21)
Diabetes Mellitus	250	Diabetes mellitus	Endocrine/metabolic	1.62 × 10^−6^	4.8	525	(2,9, 10, 11, 12, 13, 14, 15, 16, 17, 18,20, 21, 22, 23, 24,26, 27, 28,30,61, 62, 63, 64)
Bronchitis	497	Bronchitis	Respiratory	2.15 × 10^−6^	4.7	98	(12,16,20)
Dementia	290.1	Dementias	Mental disorders	1.55 × 10^−5^	4.3	78	(9,11,12,15,20,32)
COPD	495.1	Chronic obstructive asthma	Respiratory	.002132	3.1	63	(2,10,12,13,15,17,20,23,26,28,30,60, 61, 62)

COPD, chronic obstructive pulmonary disease; EHR, electronic health record.

### Identifying Phenotypes Associated With Genetic Risk of Schizophrenia

**Figure 2:**
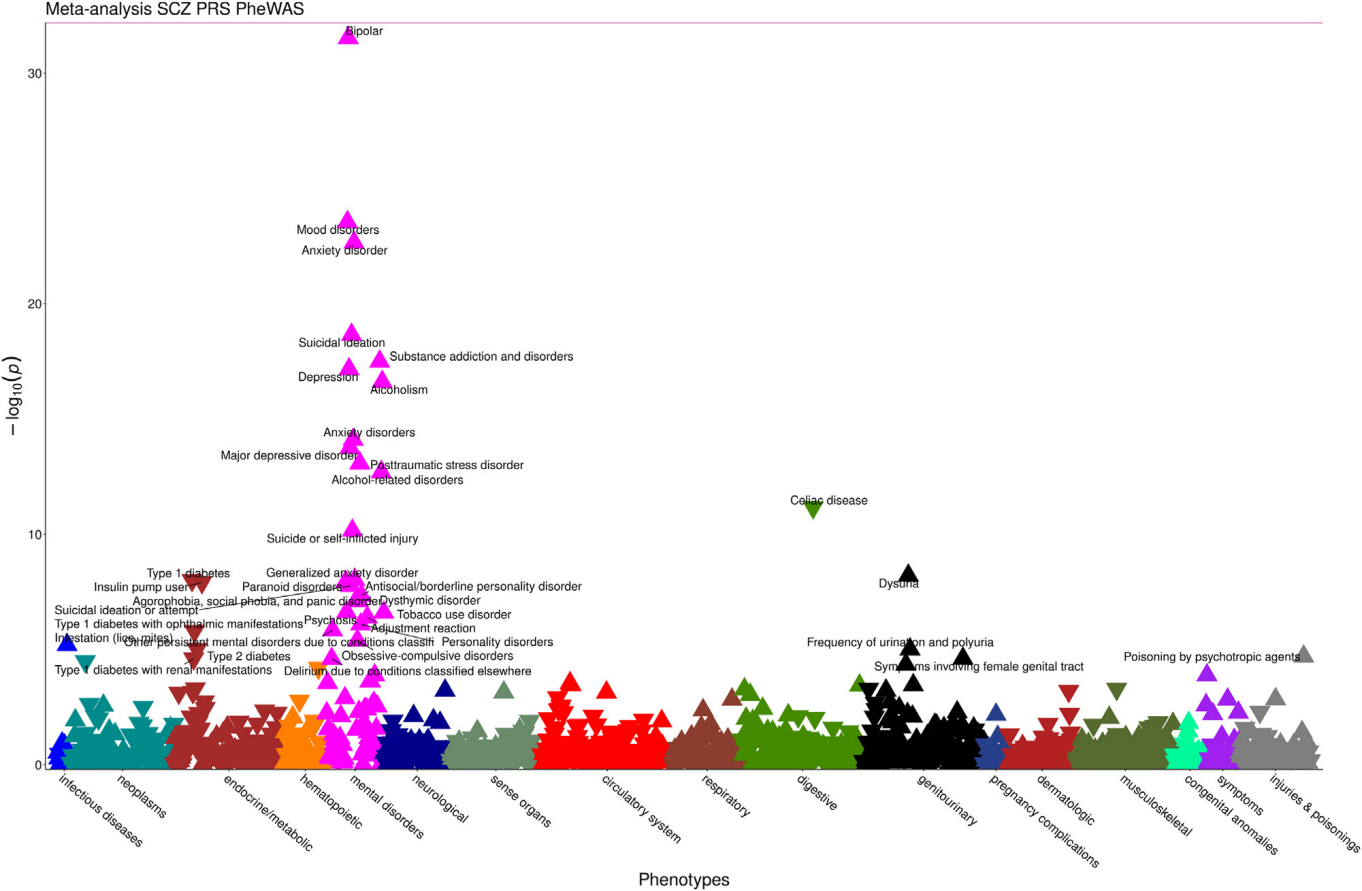
Bonferroni-significant schizophrenia (SCZ) polygenic risk score (PRS) meta-analysis associations. The x-axis is phecodes grouped by category. The y-axis is the negative log_10_ of the *p* values for the phenotypes associated with the schizophrenia PRS. Labeled triangles represent phenotypes that showed significant association after Bonferroni correction, with either positive (upright triangle) or negative (inverted triangle) direction of effect. PheWAS, phenome-wide association study.

Next, we sought to identify which phecodes were significantly associated with the schizophrenia PRS among patients of European ancestries (Table S4 in Supplement 2; Figures S3, S4 in Supplement 1), where we had sufficient sample size, in biobanks at VUMC (*n* = 63,923) and MGB (*n* = 25,698). Schizophrenia cases were removed to isolate genetic effects independent of diagnosis. Including the patients with schizophrenia in the PRS PheWAS only resulted in 6 additional associations of psychiatric disorders already known or represented in the PheWAS (Figures S3, S7 in Supplement 1). Therefore, the PRS PheWAS with patients with schizophrenia excluded was ultimately used to reduce bias. Results were combined using a fixed-effect inverse variance-weighted meta-analysis (see Methods and Materials). After Bonferroni correction for 1586 tests (*p* < 3.15 × 10^−5^), there were 38 significant schizophrenia PRS-associated phenotypes (Figure 2). As with the EHR comorbidities, the majority were psychiatric conditions such as bipolar disorder, anxiety disorder, and suicidal ideation. There were also a variety of medical illness associations such as lice infestation, dysuria, viral hepatitis C, HIV, and poisoning by psychotropic agents. Despite using diverse ancestry GWAS summary statistics and PRS-CSx (Supplemental Methods in Supplement 1), no significant PRS associations were identified in the African ancestry sample (Figures S5, S6 in Supplement 1).

### Identification of Comorbidities With Significant Absence of Genetic Contribution

Overall, the EHR-based comorbidities were significantly correlated with the PRS associations (*r* = 0.55, *p* = 1.29 × 10^−118^) (Figure S8 in Supplement 1). Similar correlations were seen when comorbidities and PRS associations were compared across sites to remove sample dependence: VUMC comorbidities with the MGB PRS associations (*r* = 0.46, *p* = 8.56 × 10^−77^) and the MGB comorbidities with the VUMC PRS associations (*r* = 0.46, *p* = 5.31 × 10^−68^). We were primarily interested in identifying phecodes with significant comorbidity but a lack of any shared genetic overlap with schizophrenia. These phenotypes represent those enriched for where common genetic variation is not a strong driver of comorbidity, implicating other potential contributors such as environmental influences, medication effects, or rare genetic variation (39). For each of the 65 significant EHR comorbidities that also had PRS results, the distributions of the PRS for cases and controls were compared using a TOST equivalence test to determine whether they were significantly equivalent (i.e., significant absence of a difference in the PRS between cases and controls) (see Methods and Materials). The equivalence test provides a statistical method for rejecting the null hypothesis that 2 distributions differ beyond the bounds of a small effect size and therefore can provide statistical support for the distributions being considered equivalent (65). In other words, the TOST procedure allows us to say that the study was sufficiently powered to detect effects of a certain size, but no such effects were seen.

After correction for multiple testing, 36 comorbidities were considered significantly equivalent (Table 2; Table S5 in Supplement 2). Included in this set were 11 phenotypes where the PRS association was significant after correction, but the effect size was small enough to still be considered significantly equivalent. For example, tobacco use disorder is the most significant result from the TOST analysis (*p* = 5.80 × 10^−74^), reflecting the small effect size of the PRS association (variance explained = 0.054%); however, because it is a common phenotype, it is significant after multiple testing correction in the PRS association (*p* = 2.43 × 10^−7^). This group also included type 2 diabetes, where the schizophrenia PRS was significant but associated with decreased risk. Requiring that the PRS association was not significant after multiple testing correction yielded 25 phenotypes, 15 of which had a PRS association *p* value > .05. In other words, these 15 phenotypes were significantly comorbid with schizophrenia and lacked any discernable difference in the schizophrenia PRS between cases and controls despite being well-powered to detect one. This set was enriched for those with prior evidence of nongenetic causes including from medication (e.g., torsion dystonia, convulsions, tachycardia) and lifestyle implications of schizophrenia including reduced hygiene (diseases of the nail) or smoking (bronchitis) (66, 67, 68, 69, 70, 71, 72, 73, 74, 75). Notably, 3 phenotypes showed significant PRS association but were not significantly comorbid with schizophrenia (*p* > .05). This included dysuria, frequency of urination and polyuria, and symptoms involving the female genital tract.

**Table 2:** Significant TOST Results

Phecode	Category	Phenotype	TOST *p* Value^a^	Meta PRS Beta	Meta PRS *p* Value	SCZ PRS VE, %
333.4	Neurological	Torsion dystonia	1.39 × 10^−6^	0.005	9.45 × 10^−1^^b^	0.002
345.1	Neurological	Epilepsy	9.09 × 10^−17^	−0.008	8.46 × 10^−1^^b^	0.000
703	Dermatologic	Diseases of nail NOS	5.74 × 10^−11^	−0.022	7.03 × 10^−1^^b^	0.010
333	Neurological	Extrapyramidal disease and abnormal movement disorders	7.31 × 10^−7^	−0.042	5.52 × 10^−1^^b^	0.047
427.7	Circulatory System	Tachycardia NOS	7.46 × 10^−61^	0.018	3.32 × 10^−1^^b^	0.003
368.9	Sense Organs	Subjective visual disturbances	4.37 × 10^−8^	−0.056	2.93 × 10^−1^^b^	0.011
345.3	Neurological	Convulsions	7.54 × 10^−38^	0.032	1.97 × 10^−1^^b^	0.008
368	Sense Organs	Visual disturbances	2.38 × 10^−18^	0.051	1.84 × 10^−1^^b^	0.016
496.21	Respiratory	Obstructive chronic bronchitis	3.32 × 10^−16^	0.053	1.73 × 10^−1^^b^	0.023
345	Neurological	Epilepsy recurrent seizures convulsions	5.73 × 10^−26^	0.050	1.35 × 10^−1^^b^	0.019
819	Injuries and Poisonings	Skull and face fracture and other intercranial injury	2.28 × 10^−10^	0.068	1.11 × 10^−1^^b^	0.024
979	Injuries and Poisonings	Adverse drug events and drug allergies	2.34 × 10^−25^	0.048	8.88 × 10^−2^^b^	0.021
291.8	Mental Disorders	Alteration of consciousness	5.02 × 10^−23^	0.054	7.80 × 10^−2^^b^	0.024
292	Mental Disorders	Neurological disorders	3.67 × 10^−21^	0.086	6.95 × 10^−2^^b^	0.000
497	Respiratory	Bronchitis	3.51 × 10^−13^	0.074	6.39 × 10^−2^^b^	0.019
349	Neurological	Other and unspecified disorders of the nervous system	1.46 × 10^−4^	0.135	1.15 × 10^−2^	0.059
250	Endocrine/Metabolic	Diabetes mellitus	5.25 × 10^−31^	−0.104	8.24 × 10^−3^	0.053
292.4	Mental Disorders	Altered mental status	3.42 × 10^−42^	0.060	7.72 × 10^−3^	0.036
338.2	Neurological	Chronic pain	1.56 × 10^−55^	0.055	7.55 × 10^−3^	0.026
290	Mental Disorders	Delirium dementia and amnestic and other cognitive disorders	6.40 × 10^−7^	0.229	5.80 × 10^−3^	0.210
496.2	Respiratory	Chronic bronchitis	3.44 × 10^−8^	0.161	4.26 × 10^−3^	0.151
315	Mental Disorders	Developmental delays and disorders	3.75 × 10^−4^	0.138	2.91 × 10^−3^	0.071
290.1	Mental Disorders	Dementias	4.37 × 10^−9^	0.164	2.79 × 10^−4^	0.153
306	Mental Disorders	Other mental disorder	1.79 × 10^−5^	0.227	2.43 × 10^−4^	0.203
313.1	Mental Disorders	Attention deficit hyperactivity disorder	2.41 × 10^−4^	0.160	1.30 × 10^−4^	0.068
290.2	Mental Disorders	Delirium due to conditions classified elsewhere	5.17 × 10^−9^	0.159	2.51 × 10^−5^	0.123
250.2	Endocrine/Metabolic	Type 2 diabetes	2.95 × 10^−60^	−0.075	9.72 × 10^−6^	0.045
290.3	Mental Disorders	Other persistent mental disorders due to conditions classified elsewhere	2.45 × 10^−4^	0.250	1.46 × 10^−6^	0.344
318	Mental Disorders	Tobacco use disorder	5.80 × 10^−74^	0.083	2.43 × 10^−7^	0.054
300.4	Mental Disorders	Dysthymic disorder	7.72 × 10^−5^	0.207	6.89 × 10^−8^	0.203
300.11	Mental Disorders	Generalized anxiety disorder	3.69 × 10^−5^	0.197	9.36 × 10^−9^	0.177
317	Mental Disorders	Alcohol-related disorders	1.45 × 10^−4^	0.307	1.98 × 10^−13^	0.431
296.22	Mental Disorders	Major depressive disorder	2.01 × 10^−21^	0.159	1.75 × 10^−14^	0.165
300	Mental Disorders	Anxiety disorders	1.72 × 10^−4^	0.343	7.86 × 10^−15^	0.290
300.1	Mental Disorders	Anxiety disorder	4.22 × 10^−18^	0.180	2.23 × 10^−23^	0.242
296	Mental Disorders	Mood disorders	3.33 × 10^−5^	0.316	2.89 × 10^−24^	0.487

NOS, not otherwise specified; PRS, polygenic risk score; SCZ, schizophrenia; TOST, two one-sided tests; VE, variance explained.

a Significant (*p* < .00065).

b Nonsignificant SCZ PRS (*p* > .05).

## Discussion

Comorbidities are common among patients with psychiatric disorders (76) and have been associated with poor outcomes that often require lengthy hospital admissions and additional monitoring from one or multiple specialists (77). Here, we sought to identify schizophrenia comorbidities that are largely unrelated to shared genetic influences, leaving other causes that will be enriched for modifiable (e.g., treatment, environment) outcomes. Identifying this set of comorbidities provides the opportunity for intervention to be administered earlier and more broadly because we know that the primary driver of this comorbid relationship is not shared common genetic risk for schizophrenia. We calculated phenome-wide comorbidity using EHR data from 2 major health care systems and demonstrated consistency across institutions and identified 77 significant comorbidities for schizophrenia. When comparing the EHR-based comorbidities to the phenotypes associated with the schizophrenia PRS, we identified phenotypes that were significantly comorbid but lacked evidence of shared genetic influences. While we do not expect all of these phenotypes to be currently modifiable (e.g., developmental delay), results were enriched for phenotypes linked to other drivers, which validates the approach of using comorbidities and genetic associations to identify comorbid phenotypes that are enriched for modifiable causes. At the same time, these results point to other potentially modifiable comorbidities such as tobacco use disorder and type 2 diabetes.

Our EHR-based comorbidity profiles of schizophrenia showed highly significant consistency across 2 independent health care institutions, demonstrating the overall robustness of our comorbidity measure (Pearson’s *r* = 0.79, for all phecode pairs and *r* = 0.85 for schizophrenia specifically). Among schizophrenia comorbidities that had been described previously in multiple studies, we identified 25 of the 59 conditions, a proportion that is significantly greater than expected by chance. These included 18 of the 19 psychiatric conditions and 7 of the 40 medical comorbidities that had previously been reported. The difference in overlap of the EHR-based comorbidities with the literature between the psychiatric and medical phenotypes potentially results from several causes. These include the temporal and specialized care of patients documented in the EHR. Most patients do not receive care for all conditions at the same institution throughout their lives. Because care was provided for schizophrenia, it is more likely that care included psychiatric care that included documentation of comorbid psychiatric conditions. However, the extent to which other medical care was provided and for what duration is highly variable and could reduce the power to identify comorbid conditions that occur much later in life or through providers seen at different institutions. Furthermore, our comorbidities were calculated within a hospital population with high rates of comorbidity overall, potentially making it harder to observe significant comorbidity for highly prevalent medical conditions such as heart disease and obesity. Nevertheless, psychiatric and medical comorbidities showed significant enrichment independently. We observed 45 EHR-based comorbidities that lacked support in the literature, presenting an interesting set of comorbidities that are less well-studied or have not been identified previously.

The frequent co-occurrence of phenotypes is often matched by shared underlying genetic risk, but there are exceptions. Factors such as unhealthy behavior (e.g., sedentary lifestyle, poor diet) could be a byproduct of the disease while environment and treatment can also influence comorbidities without genetic influence (39). Under these conditions, comorbidities may present without any genetic overlap, indicating an opportunity to identify a modifiable cause. Here, we leveraged EHR-based comorbidities with PRS association to identify those exact scenarios for schizophrenia, i.e., where schizophrenia significantly co-occurred with another phenotype but there was a significant lack of difference in schizophrenia PRS distributions between cases and controls. We identified 15 phenotypes without a nominally significant PRS association including adverse drug events, as well as recurrent seizures, extrapyramidal disease and abnormal movement disorder, tachycardia, and torsion dystonia, which have all been previously linked to antipsychotic treatment (66, 67, 68,70, 71, 72), thus validating our hypothesis. Environmental influences are also apparent, such as for bronchitis, which is influenced by the high rate of smoking in patients with schizophrenia (73,74). These results provide validation that this approach is enriching for comorbidities with other, potentially modifiable, noncommon genetic causes and could also provide valuable novel insights when applied to other phenotypes. Importantly, there is a set of phenotypes with highly significant equivalence and significant PRS association. This is a product of well-powered PRS analyses but very small effect sizes. Therefore, interpretation is limited in the case of less heritable phenotypes. Even for the most significant PRS associations among this set, the effect sizes are quite small (variance explained < 0.5%), potentially pointing to nongenetic causes as primary drivers of comorbidities. This set includes several interesting phenotypes such as tobacco use disorder, diabetes, and dementias, where prior work has implicated genetic sharing and similar etiologies (78, 79, 80, 81, 82, 83). Our results suggest that the genetic risk of schizophrenia is not the primary contributing factor to these comorbidities, supporting other potential hypotheses and with potential for intervention. In fact, these results are consistent with other studies in which the genetic risk of schizophrenia has been associated with lower risk of diabetes despite very high comorbidity, which suggests that a reasonable case can be made for other contributors such as medication (35,37,84).

There are several limitations of this work that should be noted. The first is the use of phecodes as the diagnostic measure for schizophrenia and its comorbidities. ICD codes are designed for clinical documentation and billing purposes, not for research. To reduce the potential for misclassification, we used phecodes that group related ICD codes together, and we required cases to have at least 2 phecodes recorded on independent dates. However, these codes can vary by clinical practice, and because they are captured over time, they also represent the evolution of a patient’s clinical presentation. For example, bipolar disorder and schizophrenia are clinically independent diagnoses based on DSM-5, but bipolar disorder is the most significant schizophrenia comorbidity across both sites, representing potential misdiagnosis during clinical presentation over time. An alternate approach of defining cases by the prevailing diagnosis was recently evaluated in a MVP (Million Veteran Program) study and was found to reduce diagnostic overlap. However, the investigators also found that a 2+ code definition accurately captured 95% of cases and had the best overall balance of sensitivity vs. specificity (37). Importantly however, these phecodes represent real-world clinical data, and the strength of comorbidity using this phecode definition was highly consistent across 2 independent institutions located in different parts of the country. The PRS associations are dependent on the genetic component captured by common variation tested in a GWAS. Any other genetic factors including rare variants could still be driving the comorbidities seen here. The schizophrenia GWAS is well-powered, identifying hundreds of genome-wide significant loci with a heritability estimate of 24% (47). A GWAS with more statistical power could identify additional PRS associations, but the effects are likely to be small. Comorbid phenotypes with no genetic support at all would be identified by this approach and would not be specific to schizophrenia. Due to limited genetic sample size, we were not powered to perform this study in any non-European ancestries. This is unfortunate given the many racial disparities inherent in health care such as variation in treatment prescription, monitoring of adverse drug effects, social disadvantages, and negative connotations attributed to the patient by providers that bias interpretation of results (85, 86, 87, 88, 89, 90). Understanding differences in modifiable comorbidities across ancestries is an important future direction for research. While our approach identified phenotypes that fit our expectations, it does not explicitly point to whether those comorbid phenotypes occur before or after the diagnosis of schizophrenia and therefore provide insight into what may be potential causes. Efforts to establish more robust inferences about causal pathways are necessary. EHR data can be helpful for this process, but substantial consideration of its limitations will be needed to account for data fragmentation and incomplete time course.

### Conclusions

This work provides substantial evidence of the consistency and robustness of comorbidity defined using EHR data based on comparison across multiple health care institutions, validation from the literature, and consistency with genetic association. We presented an approach that integrates EHR-based comorbidities with PRS-based genetic association to identify comorbidities without genetic overlap. These phenotypes present potentially modifiable outcomes driven by other factors such as behavior, treatment, or environment. In schizophrenia, we showed multiple examples of this approach working and highlighted other phenotypes that are worthy of further work to identify the causal path. Overall, this work provides a path to better understanding comorbidities that may be more easily modified to improve outcomes.

## Supplementary Material

Key Resources Table
